# Mechanistic insights into the chemistry of compound I formation in heme peroxidases: quantum chemical investigations of cytochrome *c* peroxidase†

**DOI:** 10.1039/d2ra01073a

**Published:** 2022-05-23

**Authors:** Mohamed M. Aboelnga

**Affiliations:** Chemistry Department, Faculty of Science, Damietta University New Damietta 34517 Egypt aboelng@uwindsor.ca

## Abstract

Peroxidases are heme containing enzymes that catalyze peroxide-dependant oxidation of a variety of substrates through forming key ferryl intermediates, compounds I and II. Cytochrome *c* peroxidase (Ccp1) has served for decades as a chemical model toward understanding the chemical biology of this heme family of enzymes. It is known to feature a distinctive electronic behaviour for its compound I despite significant structural similarity to other peroxidases. A water-assisted mechanism has been proposed over a dry one for the formation of compound I in similar peroxidases. To better identify the viability of these mechanisms, we employed quantum chemistry calculations for the heme pocket of Ccp1 in three different spin states. We provided comparative energetic and structural results for the six possible pathways that suggest the preference of the dry mechanism energetically and structurally. The doublet state is found to be the most preferable spin state for the mechanism to proceed and for the formation of the Cpd I ferryl-intermediate irrespective of the considered dielectric constant used to represent the solvent environment. The nature of the spin state has negligible effects on the calculated structures but great impact on the energetics. Our analysis was also expanded to explain the major contribution of key residues to the peroxidase activity of Ccp1 through exploring the mechanism at various *in silico* generated Ccp1 variants. Overall, we provide valuable findings toward solving the current ambiguity of the exact mechanism in Ccp1, which could be applied to peroxidases with similar heme pockets.

## Abstract 2

Discerning the feasibility of a no-water peroxidase mechanism in the doublet spin state irrespective of the environment surrounding the heme pocket.

## Graphical abstract

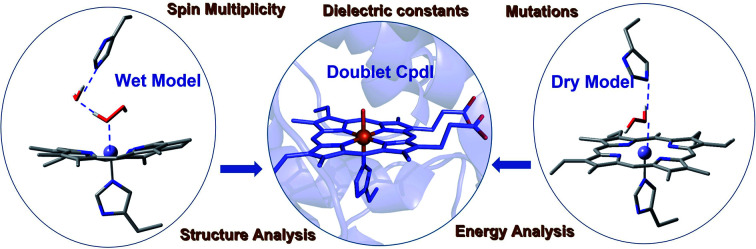

## Introduction

Heme enzymes are crucial in biology and exist in almost all living organisms.^1^ Exploiting the catalytic redox power of the iron-containing heme cofactor, they catalyze a wide range of biological redox reactions.^2^ Heme peroxidases are the most abundant heme enzymes and activate hydrogen peroxide to oxidize a variety of substrates.^3^ A defining feature across the whole heme enzyme family is the formation of key ferryl intermediates (compound I), involving a porphyrin π-cation radical, during their catalytic cycle.^4–6^ Identifying the chemical nature of the oxyferryl bond in compound I has been extensively studied but its precise chemical identity remains a matter of increasing debate.^4,5,7–10^

**Scheme 1:**
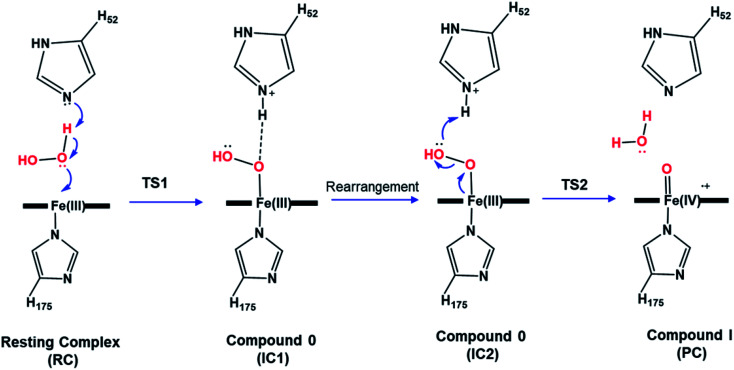
Proposed catalytic mechanism of Cpd I formation in heme peroxidases.

The most widely accepted catalytic pathway for the formation of compound I (Cpd I) by peroxidases is the Poulos–Kraut acid–base mechanism.^11^ In this mechanism, the conserved His residue in the distal site acts as an acid–base catalyst that promotes heterolytic cleavage of the O–O bond of the peroxide group.^12^ The resting-state ferric iron binds H_2_O_2_ and the reaction is triggered by an electron transfer from the iron toward the substrate leading to the formation of compound I, a ferryl intermediate with an unprotonated iron–oxo double bond (Fe^IV^=O).^13^ Previous computational studies explored this mechanism in various peroxidases and reported a two-step mechanism in which the heterolytic cleavage of the O–O bond is the rate-limiting step.^12–15^ According to these studies, the first step to form the ferric-hydroperoxide (Fe^III^–OOH) intermediate (Cpd 0) takes place through proton abstraction from the bound peroxide in Fe^III^–O_2_H_2_ by the nearby His acid–base catalyst (IC1), Scheme 1. This step is followed by a structural rearrangement of the imidazole side chain of the newly protonated His generating a second conformation of Cpd 0 (IC2), in which the His residue is hydrogen-bonded with the distal OH group of Fe–OOH, Scheme 1. Subsequently, the protonated His residue releases its proton to the distal OH, promoting heterolytic cleavage of the O–O bond together with formation of a water molecule.^16^ However, other theoretical studies support the proposal that a nearby water molecule may assist proton transfer from H_2_O_2_ to the His acid–base catalyst.^17,18^ This proposal is in consistent with the long distance reported between the catalytic His and H_2_O_2_ and the presence of crystalized water molecules around the ferryl oxygen.^2,12^ According to these studies, the incorporation of a bridged water molecule in the first step of the mechanism lowered the activation barrier.^12,17^ Thus, in contrast to the initial no-water (dry) pathway, an water-mediated (wet) mechanism has been proposed for peroxide activation by peroxidases.^2,17,18^ Indeed, water-assisted mechanism is well-known approach in facilitating the reaction in other fundamental enzymes.^19,20^

Cytochrome *c* peroxidase (Ccp1) served for decades as a benchmark biochemical model for understanding the chemical and structural properties of other peroxidases.^6^ Its principal function is to eliminate the toxic H_2_O_2_ from the cell with the assistance of its redox partner cytochrome c (Cc).^21^ The ferrous heme in Cc is approximately 20 Å away from the ferric heme of peroxidase and the combined Ccp1–Cc complex has been considered as a paradigm for probing long-range electron transfer in biology.^6,22^ Despite the presence of a universal heme pocket amongst heme-peroxidases, Ccp1 known to stand out, with a well-characterized tryptophan (W191) π-cation radical compound I.^23^ This distinctive electronic feature of Ccp1's ferryl intermediate is attributed mainly to the electrostatic effect of the surrounding environment of the critical W191 residue.^24^ In fact, the nearby methionine residues (M230 and M231) are found to be vital for the stabilization of the W191 radical through S-aromatic interactions.^25^ The absence of these residues together with the presence of a cation binding site in ascorbate peroxidase (APX) resulted in a porphyrin π-cation radical instead as Cpd I.^24,26^ The W191 residue in Ccp1 together with the nearby redox-active (Trp, Met and Tyr) residues play an important role in the hole hopping pathway from the heme pocket to Ccp1's surface and eventually to Cc.^27^

**Fig. 1:**
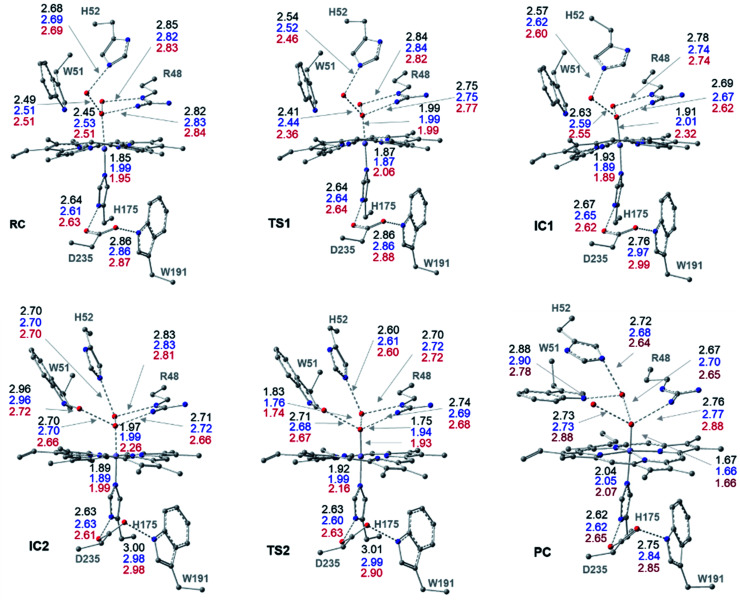
Cluster structures along the wet-mechanism pathway of Cpd I formation, with selected distances between heavy atoms (Å), for three different spin states: doublet (black), quartet (blue) and sextet (red). Geometries are optimized with UB3LYP-D3BJ/6-31G(d,p).

The axial H175 is conserved amongst heme peroxidases and its ligation to the Fe centre is paramount to the redox activity of the system.^28–30^ Its strong H-bond interaction with the adjacent D235 residue is vital to induce imidazole character and thus lower the redox potential of the iron centre.^31,32^ Mutagenesis studies demonstrated the severe modulation of peroxidase activity upon changing the chemical identity of the axial ligand.^32–34^ Moreover, the proximal triad H-bond interaction between the conserved D232, W191 and H175 residues, Fig. 1, is found to play a fundamental role in correctly positioning W191 for the hole hoping pathway and thus efficient peroxidase activity of Ccp1.^31,35–38^

Various key intramolecular interactions in both the proximal and distal sites around the heme are central for the system stability and reactivity.^39–41^ Three residues located in the distal site of the heme, namely W51, H52 and R48, are within close proximity to influence, *via* strong H-bond interactions, the proper positioning of H_2_O_2_ near the Fe(iii) ion to trigger the peroxidase reaction.^42^ H52 residue is ideally oriented to act as an acid–base catalyst and its mutation leads to a significant drop in the catalytic rate of the reduction process.^2,40,43,44^ Both R48 and W51 residues feature side-chain geometries consistent with H-bond interaction with the oxyferryl oxygen in compound I and thus help stabilize it.^5,45,46^

Recent all-atom molecular dynamics (MD) simulations conducted by our group on Ccp1 with H_2_O_2_ bound to the ferric iron suggest that the catalytic H52 residue lies close to H_2_O_2_, with an average distance *r*(_H52_N⋯HO) = 2.32 Å consistent with strong H-bond interactions.^47^ This observation brings support for the dry mechanism of Cpd I formation in Ccp1, and not the water-assisted one proposed earlier.^2,17^ This is in agreement with previous MD investigations of horseradish peroxidase (HRP) that demonstrated the very low probability for a catalytic water molecules to bridge the catalytic His residue and H_2_O_2_.^15,17,46^ In addition to other quantum chemistry calculations (QM) on different heme peroxidases that suggested a dry mechanism for the formation of the Fe–hydroperoxide containing complex (Cpd 0).^13,14^ This warrants further investigation of the feasibility of the water-assisted *vs.* dry mechanism for the formation on Cpd I in heme peroxidases with Ccp1 as a case study. Accordingly, we report in this manuscript the results of QM calculations of the dry and wet mechanisms for Cpd I formation in Ccp1. Careful considerations were given to the possible spin states of each model which results in probing the catalytic mechanism for a total of 6-different models, our objective is to better characterize the preferable pathway and shed light onto the role of essential residues in both the formation and stability of the high-valent oxyferryl intermediate, Cpd I.

Detailed comparison between the structural and electronic features of Cpd I at different spin states has been also conducted for both wet and dry models. We obtained a kinetically preferred pathway in which we underlined various structural parameters that align with experimental ones. Moreover, the preferred mechanism was explored at various *in silico* generated variants for key residues to further clarify their precise roles in the mechanism, the geometry, and the electronic behaviour of Cpd I. Deep investigations were conducted to analyze all the obtained structures during the mechanism for each model. Our findings explain the impact of each point of mutation on the structures collected during the reaction mechanism. Due to the conserved nature of the heme pocket amongst heme-peroxidases, the obtained findings from our extensive investigation could be universal amongst them.

## Computational methodology

QM cluster calculations have been successfully applied to model numerous catalytic reactions.^48–52^ The cluster model used herein is obtained from the crystal structure (PDB ID: 3M2I resolution: 1.40 Å) of the compound I ferryl intermediate.^53^ The resting-state ferric-containing heme structure was generated by morphing the bound oxygen and the nearby oxygen into H_2_O_2_ for the dry model. A single additional water molecule was placed in a bridging position between H52 and peroxide to build the water-assisted model. Our cluster models include the heme pocket residues that have been previously suggested to directly participate in the catalytic mechanism. These crucial residues participate in the mechanism through either activating H_2_O_2_ and/or neutralizing the developing charges in the transition states and intermediates, and therefore enhance the chemical reactivity of the heme. This QM model includes the distal R48, W51 and H52 residues in addition to the proximal H175, W191 and D235 residues. All the residues are truncated at their α-carbons and these latter carbons are kept frozen to their coordinates in the crystal structure (PDB ID: 3M2I). This resulted in dry and water-assisted models with a total of 170 and 173 atoms, respectively, and an overall charge of +1, as a starting point to investigate the mechanism of H_2_O_2_ activation, Scheme 1.^54–56^ The open d-shell of the heme iron allows for various possible electronic configurations and thus different multiplicity states, namely, doublet, quartet and sextet states, have been considered for each pathway.^57,58^ The spin multiplicity is indicated as a left-hand superscript for each structure calculated throughout.

Density-functional theory (DFT) calculations are performed with the unrestricted B3LYP functional,^59–61^ which has been highly recommended for heme-system studies,^62,63^ in conjunction with the 6-31G(d,p) basis set, as implemented in Gaussian 16 (revision B.01).^64^ The D3 version of Grimme's dispersion with Becke–Johnson damping (GD3BJ) correction is used to improve the treatment of noncovalent and dispersion interactions.^65^ Spin contamination was investigated for all the optimized structures to ensure that it is not significant. Following geometry optimization, vibrational frequency analyses are performed to characterize the nature of the stationary points. Visualization of the atomic motion associated with the calculated imaginary-frequency mode was performed to connect every transition state (TS) to the corresponding intermediates on the potential energy surface. Electronic energies were further refined with a larger basis set, and the cluster environment was taken into account with the integrated effective fragment polarizable continuum model (IEFPCM) and a dielectric constant *ε* = 4 which is generally believed to represent the protein environment [IEFPCM-B3LYP-GD3BJ/6-311+G(2d,2p)].^50,51,66^ Another set of functions namely [IEFPCM-B3LYP-GD3BJ/6-311+G(2df,2p)] was conducted to calculate the energy for one full pathway to confirm the negligible impact of changing the basis set, Table S3.† The efficiency of using the current basis set for geometry optimization was confirmed to be accurate by comparison of structural results obtained for Cpd I in the three different spin states relative to more expensive basis sets (*cf.* ESI Fig. S1†). Environmental effects were further explored by repeating the calculations for Cpd 0 and Cpd I with dielectric constants *ε* = 4, 8, 16 and 78.4 (*cf.* ESI Tables S3 and S4†).

## Results and discussion

**Fig. 2:**
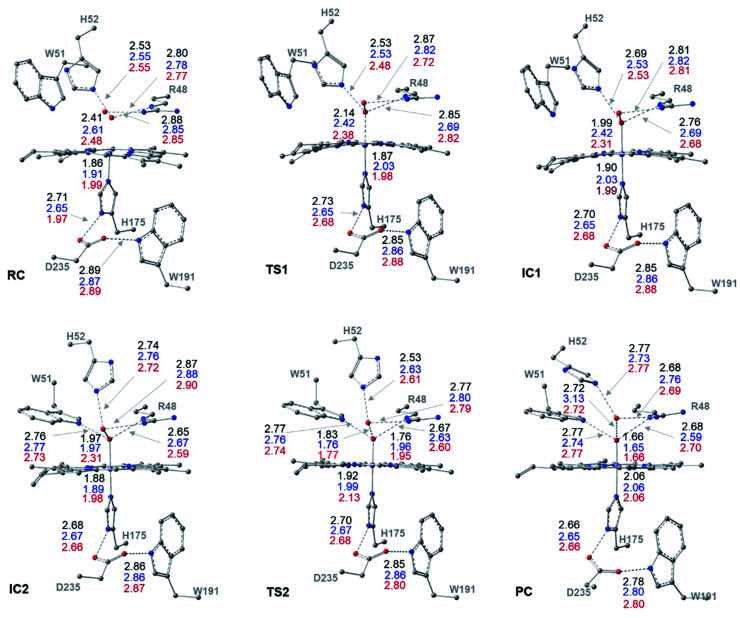
Cluster structures along the dry-mechanism pathway of Cpd I formation, with selected distances between heavy atoms (Å), for three different spin states: doublet (black), quartet (blue) and sextet (red). Geometries are optimized with UB3LYP-D3BJ/6-31G(d,p).

**Fig. 3:**
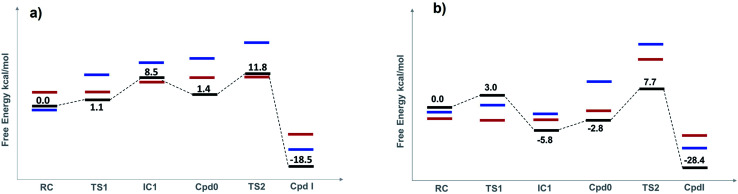
Free-energy profiles (kcal mol^−1^) for both the wet (a), and dry (b) mechanisms of Cpd I formation for three different spin states: doublet (black line), quartet (blue line) and sextet (red line). Free energies are estimated with UB3LYP-GD3BJ/6-311+G(2d,2p) with *ε* = 4 (see text for details).

The optimized structures of all intermediates and transition states along the wet and dry-mechanism pathways of Cpd I formation are shown in Fig. 1 and 2, respectively, while free energy profiles are depicted in Fig. 3 and the associated free energy values for all structures are collected in Table S1.†

### I. Wet mechanism of cpd I formation

The reaction mechanism starts from a fully optimized reactive complex (RC) in which the H_2_O_2_ is bound to the Fe(iii) ion and close to the bridged water molecule (W). From the structures in Fig. 1, the latter water molecule is properly positioned to facilitate a proton transfer from H_2_O_2_ to the nearby catalytic H52 residue. The R48 residue geometry is consistent with H-bond interactions with the two oxygens of H_2_O_2_, demonstrating its role in positioning the ligand in the catalytic distal site. The H-bond interactions between the proximal H174, W191 and D235 residues appear to be maintained regardless of the system's spin state. The RC geometries obtained in the three spin states align structurally, with ^6^RC thermodynamically the least stable. The structures of ^2^RC and ^4^RC lie very close in energy, within 0.5 kcal mol^−1^, and ^4^RC is slightly more stable, Fig. 3a.

The formation of the Cpd 0 intermediate IC1, in which the H52 residue becomes protonated, and a Fe^III^–OOH single bond is formed, takes place through the first transition state (TS1), Fig. 1. TS1 exhibits a water-assisted double proton transfer from H_2_O_2_ to the nearby H52 residue. The lowest free energy barrier for this concerted step is found to be 1.1 kcal mol^−1^ for ^2^TS1 relative to its ^2^RC, Fig. 3a. The second lowest free energy barrier is for ^6^TS1 (2.4 kcal mol^−1^ higher in energy than ^2^TS1) with has an activation energy of 2.1 kcal mol^−1^ relative to ^6^RC. It is also worth mentioning that the higher energy values of IC1 relative to its TS1 is noticed only upon adding the Gibbs energy correction. In fact, the relative energies for the obtained structures before adding the Gibbs correction are collected in Table S2† and TS1 is higher in energy than the following IC1. The structural parameters for TS1 in different spin states are very similar, and the most significant change is obtained for the length of Fe–O bond, which is 0.28 and 0.16 Å longer in both ^4^TS1 and ^6^TS1, respectively, than in ^2^TS1. Notably, the _H52_N⋯H-bond distance in ^6^TS1 is found to be 0.05 Å shorter than its value in both ^2^TS1 and ^4^TS1, Fig. 1.

The optimized geometries of ^2^IC1 and ^6^IC1 are found to be the most thermodynamically stable and pseudo-degenerate, with similar structures except for the elongation of the Fe⋯O bond by 0.41 Å in ^6^IC1 relative to ^2^IC1, Fig. 1. This intermediate encountered a structural rearrangement of the H52 imidazole side chain consistent with H-bond interactions with the distal OH of the ligated hydroperoxide group, Scheme 1 and Fig. 1, to form the other conformation of Cpd 0 intermediate (IC2). The structures of the IC2 intermediate are very similar in all three spin states but its most thermodynamically stable form is that of the doublet state. It is also worth noting that the axial Fe⋯O bond in ^6^IC2 remains the longest, with a difference of 0.29 Å relative to that for the doublet analogue (^2^IC2).

The second step of the mechanism proceeds through a proton transfer from the newly protonated H52 residue to the H-bonded distal –OH group, promoting heterolytic cleavage of the O–O bond. The lowest free energy barrier for this step is found to be 11.8 kcal mol^−1^ for ^2^TS2 relative to ^2^RC, Fig. 3a. Notably, ^4^TS2 and ^6^TS2 are found to be thermodynamically pseudo-degenerate with a single structural difference in the distance between the distal oxygen and the bound one (0.90 Å shorter in the doublet), Fig. 1 and 3a. The activation barrier for heterolytic cleavage of the O–O bond calculated here is in a good agreement with those obtained for similar peroxidases which were in the range of 9–14 kcal mol^−1^.^12,13^ This step is followed by formation of Cpd I (PC) which is found to be thermodynamically stable in all spin states, Fig. 3a. Geometries for PC in the three spin states exhibit minimal structural differences, and ^2^PC is found to be the most thermodynamically stable, Fig. 1 and 3. In these ferryl intermediates, the average Fe–O bond distance is 1.66 Å, in agreement with experimental observations,^4,5^ indicative of double-bond character (Fe^IV^=O), Fig. 1. The most considerable structural change is obtained at the distance between W molecule at the oxo-ferryl oxygen in ^6^PC that is only 0.15 Å longer than the corresponding distance in both ^2^PC and ^4^PC. While the second most significant change is at the distance of R48 with the oxo-ferryl group in ^6^PC which is 0.11 and 0.10 longer than the corresponding distances in ^2^PC and ^4^PC, respectively.

Inspection of the _H175_NH⋯^−^OOC_D235_ and _D235_COO^−^⋯HO_W191_ distances in the various structures, Fig. 1, indicates that the tight H-bond interactions between the proximal residues persist throughout the entire mechanism. Moreover, the geometry of the R48 residue suggests consistent H-bond interactions with the two oxygens of peroxide along the pathway, indicating its crucial role in the entire mechanism. Meanwhile, the W51 residue does not seem to be involved in any H-bond interaction with the distal oxygen up until IC2 formation, Fig. 1. Importantly, there are two water molecules, W molecule and the one liberated after the O–O bond cleavage, in the heme pocket of Cpd I upon Cpd I formation and both are H-bonded to the oxyferryl group, Fig. 1. This structure is consistent with the crystal structures of other ferryl intermediates which contain more than one single water molecule (PDB ID: 1ZBZ and 6P43),^10,67^ but unlike the crystal structure which was used to build the current model (PDB ID: 3M2I).^53^ In general, the free energy profile for Cpd I formation through the wet mechanism indicates the kinetic and thermodynamic preference of doublet spin state, Fig. 3a.

### II. Dry mechanism of cpd I formation

The structures of the reactive complex (RC) for the dry mechanism are found to be very similar regardless of spin state but ^6^RC is found to be the most thermodynamically stable, Fig. 3b. In all spin states, H_2_O_2_ is orientated properly with respect to both the catalytic H52 residue and the Fe(iii) centre with average _H52_NH⋯HO_H_2_O_2__ and _H_2_O_2__O⋯Fe distances of 2.50 and 2.55 Å, respectively, Fig. 2. This positioning of H_2_O_2_ illustrates the feasibility of a direct proton transfer toward the H52 residue in the first step of the reaction to access IC1 (Cpd 0), Scheme 1.

The first transition state in the dry mechanism displayed low energy barrier relative to RC regardless of the spin states and ^6^TS1 suggests a barrierless reaction, Fig. 3b. The highest free energy barrier for this step is obtained for ^2^TS1, with a value of 3.0 kcal mol^−1^, Fig. 3b. Relative to the lowest barrier for the corresponding step in the water-assisted mechanism, the Gibbs free energy for this step is only 1.4 kcal mol^−1^ higher in energy than the corresponding step in the water-assisted mechanism, Fig. 3. Thereby, the inclusion of a water molecule to bridge the proton transfer from the peroxide to the catalytic H52 did not result in significant drop in the energy barriers unlike previous suggestions.^17,18^

The IC1 and IC2 (Cpd 0 intermediates) obtained in this mechanism differ only in the orientation of the H52's side chain relative to the distal hydroxyl of Fe–OOH, Scheme 1 and Fig. 2. Except for Fe–O distances (longer in ^4^IC1 and ^6^IC1 by 0.43 and 0.32 Å relative to ^2^IC1), the optimized IC1 geometries align structurally very well in all spin states, Fig. 2. Similar structural parameters are obtained for IC2 with the single geometrical difference lying in the Fe–O bond, which elongates by 0.34 Å in ^6^IC2. The determined ^2^IC1 and ^2^IC2 are found to be the most thermodynamically stable forms of the system after PC and lie 0.3 and 2.8 kcal mol^−1^ lower in energy than ^2^RC, respectively, Fig. 3b. Interestingly, ^2^IC1 is found to be more stable stationary point than ^2^RC. In fact, this latter observation is consistent with earlier studies on Ccp1 and APX that showed the better stability of the ferric hydroperoxide intermediate as a result of the high acidity of Fe–OOH_2_.^12,14^ Moreover, a barrier free process for the proton transfer from the distal Fe–OOH_2_ to the catalytic His has been obtained in these earlier studies.

The newly protonated H52 residue is within tight H-bond distance with the distal Fe–OOH group with *r*(_H52_N⋯O) = 2.74 Å in ^2^IC2 triggering the hydrolytic cleavage of the O–O bond as in TS2, Scheme 1 and Fig. 2. Despite structurally analogous TS2 for the three spin states, our obtained data demonstrate that ^2^TS2 is the most kinetically preferable with an activation energy of 7.7 (10.5) kcal mol^−1^ relative to the corresponding ^2^RC (^2^IC2), Fig. 3b. In comparison to the obtained energy barriers for the analogous step in the wet mechanism, the energy barrier for the current step relative to ^2^IC1 (a more stable intermediated the ^2^RC) is 1.3 kcal mol^−1^ lower and thus more kinetically preferable. Then, the collapse of latter TS resulted in the generation of a thermodynamically stable Cpd I by splitting of a water molecule associated with the formation of the unprotonated high-valent iron–oxo double bond (Fe^IV^=O) bond, Fig. 2. The average iron–oxo distance is 1.66 Å, indicative of double bond character in agreement with generally accepted bond-order to bond-distance values.^5,68^ The calculated Cpd I PC geometries are almost identical in the three spin states but ^2^PC is found to be the most thermodynamically stable, lying 28.4 (28.1) kcal mol^−1^ lower in free energy than ^2^RC (^2^IC1), in agreement with previous studies, Fig. 3b.^14,69^ The only observed structural difference between Cpd I in the three spin states is the position of the leaving water molecule relative to the proximal oxygen in Fe^IV^=O in ^4^PC, and thus affects its interactions with the nearby R48 residue, Fig. 2.

The role of the R48 residue is further clarified through positioning in conformation consistent with tight H-bond distances with the oxygen atoms of the H_2_O_2_ ligand in all the reaction steps regardless of the spin states, Fig. 2. This observation demonstrates its role in properly orienting the H_2_O_2_, neutralizing the developed charges in TSs and finally facilitating the formation of Cpd I. Meanwhile, W51 residue is within tight H-bond distance with the axial Fe–O group but only upon the IC2 formation and remained until the generation of Cpd I at all the spin states, Fig. 2. Notably, the distal H-bonded network in the proximal site (H175, W191, D235) and around the oxy-ferryl group in Cpd I is in a good agreement with the crystalized ferryl-intermediated Cpd I, Fig. S2.† In this structure, the R48 and W51 residues and the nearby water molecule is within tight H-bond distances with the oxyferryl group whereas H52 residue is H-bonded to the departed water molecule.

**Fig. 4:**
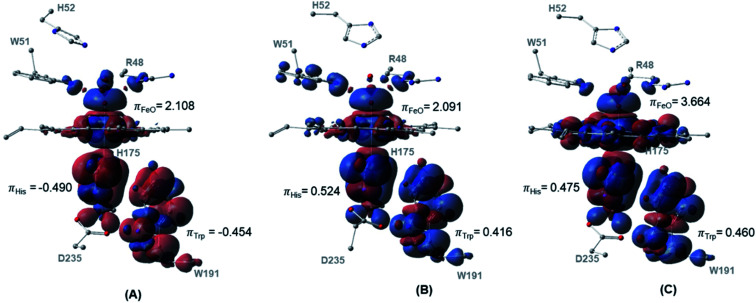
Spin density surfaces together with values for the three major spin contaminated components of (A) doublet (B) quartet (C) sextet. Calculated with UB3LYP-D3BJ/6-31G(d,p).

The spin density distributions for Cpd I in all three spin states of the dry model are shown in Fig. 4. The major contributing components in the spin density are found to be the oxyferryl group, and more importantly the H175 and W191 residues. It is observed from both the density surface that the spin density of the radical is localized on both W191 and H175 residues. The closer distance together with the π-stacking interaction between H175 and W191 residues resulted in mixing of their π-orbitals and thereby equal spin density distribution.^70^ Across all the three spin densities, the spin contamination on these two proximal residues is almost similar regardless of the spin states, Fig. 4. The major increase in the spin density is found to be located on the oxyferryl group of the sextet spin state. The antiferromagnetic coupling between the alpha radicals on oxyferryl group and the radical over the π orbitals of W191 and H175 residues generates doublet state, whereas the ferromagnetic coupling between them resulted in the quartet state, Fig. 4.

Previous study concluded the dependency of the spin-energy splitting in peroxidases on the external perturbation.^71^ Thereby, to confirm the thermodynamic preference of Cpd 0 and Cpd I in the doublet spin states in various environments, we reoptimized the structures for both Cpd 0 and Cpd I for the three spin states at four different dielectric constants to represent four surrounding environments, Fig. S2.† The free energy values for Cpd 0 and Cpd I structures are collected in Table S2† while their corresponding geometries are depicted in Tables S3 and S4.† Our calculations demonstrate the negligible structural perturbation of the geometries in the studied environments. In addition, upon conducting energetic comparison between the Cpd 0 and Cpd I structures in different spin states, the doublet spin state for both intermediates dominate the other spin states supporting our initial findings. This observation also supports the proper representation of our current chemical cluster to explore the mechanism of Ccp1. In particular, the size of our chemical model aligns perfectly with the generally suggested size that is known to be less affected by the nature of the surrounding dielectric constants.^50,72,73^

### III. Studying the mechanism of cpdI formation at different *in silico* generated QM variants

To further probe the impact of various key residues and their associated interactions on the mechanism of Cpd I formation, we studied the peroxidase mechanism for eight generated Ccp1 variants. Comparative structural, energetic, and electronic analysis of each individual point of mutation relative to the corresponding values in WT Ccp1 have been conducted. The generated *in silico* Ccp1 variants include the following: W191Y, W191A, W51A, R48A, W51/W191F (to model the active site of HRP)^68^ D235E, neutral D235, D235N, and D235A. For each one of these *in silico* generated variants, we explored the mechanism of Cpd I formation starting from the first conformation of Cpd 0 (IC1) in the doublet state through a dry model, the most preferred pathway obtained at WT Ccp1, Fig. 3b. Our attempts to locate RC for most of the studied Ccp1 variants have failed and the structure of the reactive complex fell apart toward more stable intermediate (IC1) and thus the latter has considered as the reaction starting complex. All the structures along the pathway for each of the variant models (starting from IC1) have been fully characterized and the distances for various key interactions are collected in Tables S7–S10,† while their associated Gibbs free energy for chemical reactions are collected in Table 1. Overall, none of the studied Ccp1 variants resulted in considerable geometrical change in the obtained key distances relative to WT Ccp1, except for a local change nearby the point of mutations, which is in a good agreement with former experimental observation.^39,41,74–76^

**Table 1:** Gibbs free-energy (kcal mol^−1^) for the mechanisms of Cpd I formation for WT and the other studied *in silico* variants in the doublet state, estimated with UB3LYP-GD3BJ/6-311+G(2d,2p) + Δ*G* with *ε* = 4

	WT	R48A	W51A	W51F/W191F	W191Y	W191A	D235E	D235N	D235H	D235A
IC1	0.0	0.0	0.0	0.0	0.0	0.0	0.0	0.0	0.0	0.0
IC2	−2.5	3.3	0.8	1.6	−2.6	−3.8	−3.1	−3.1	−4.1	−5.6
TS2^a^	8.0 (10.5)	13.0	14.3	11.8	8.3 (10.9)	8.8 (12.6)	7.4 (10.5)	9.1 (12.2)	7.4 (11.5)	6.3 (11.9)
PC	−31.9	−22.6	−19.1	−22.0	−25.3	−25.4	−26.8	−24.5	−25.5	−27.2

a The energy values between brackets are calculated relative to IC2 in cases where it is thermodynamically more stable than IC1.

However, relative to the calculated Gibbs energy barrier obtained for WT Ccp1, the studied Ccp1 variants demonstrated higher energy barrier for the rate-limiting step, and thus lower reaction rate, that is the heterolytic cleavage of O–O bond. The maximum influence on the reaction barrier is obtained for W51A and R48A Ccp1 variants which gave energy barriers that are 3.8 and 2.5 kcal mol^−1^ higher than the barrier obtained for WT Ccp1, Table 1. This noticeable impact on the activation energies clearly indicates the essential roles of these residues in the overall Cpd I formation determined experimentally.^34,39,41,67,76^

The similar chemical electronic and chemical behaviour between the readily oxidizable tyrosine and tryptophan residues at 191 position has been shown experimentally.^74^ In agreement with that, the calculated barrier for W191Y Ccp1 is only 0.4 kcal mol^−1^ than the value of WT Ccp1. Greater impact on the energy barrier is found on the other proximal site substituted models except for D235E Ccp1. The Glu235 residue at the latter D235E Ccp1 model adopts a geometry consistent with forming H-bond interaction with both H175 and W191 residues similar to WT Ccp1 during its reaction coordinate, Table S7–S10.† Thereby, a similar reaction barrier to the WT Ccp1 is obtained for this variant. Except for variants related to the distal site residues, the 2nd conformation of Cpd 0 (IC2) is thermodynamically more stable than the corresponding 1st conformation (IC1). Furthermore, all the energy profiles possess a thermodynamically stable product complex (indicative of Cpd I) relative to their IC1, Table 1.

**Fig. 5:**
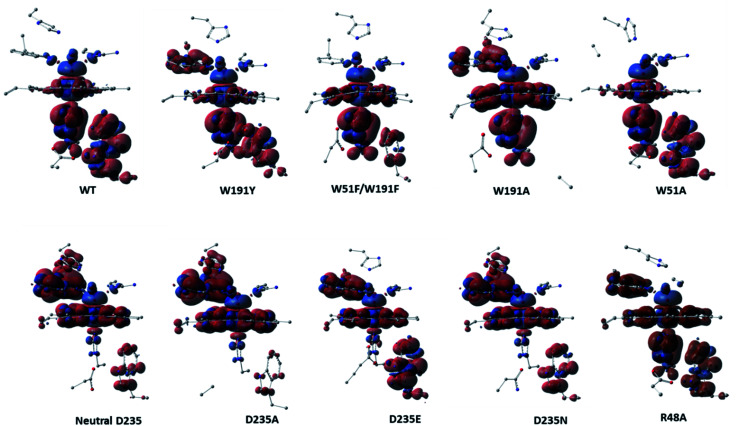
Spin density surfaces together with values for the three major spin contaminated components of WT Ccp1 and its *in silico* generated variants. Calculated with UB3LYP-D3BJ/6-31G(d,p).

Analysis of the spin density distributions for Cpd I for all *in silico* Ccp1 variants were conducted and provided in Fig. 5 and their values are collected in Table S11.† Relative to WT Ccp1 which shows the major contribution of W191 in the overall spin contamination, W191Y, W51A and R48A Ccp1 variants display a similar electronic manner. However, W191A and all mutations at D235 position adopt porphyrin π-cation radical with minimal spin density over 191 positions but on W51 residue instead. W51F/W191F Ccp1 doublet mutant, that resembles the heme pocket of HRP, demonstrates a porphyrin π-cation radical in consistence with experimental observation.^2^ The geometry of E235 at D235E resulted in a spin contamination over W191 residue similar to WT Ccp1, whereas N235 residue at D235N, which affords only a single and weaker H-bond interaction with the nearby H175 residue, shows less spin density accumulated over W191. Overall, changing the net of interactions around the essential W191 display a great impact on the electronic behaviour of the system.

## Conclusions

Extensive DFT calculations on a cluster model containing the heme pocket of Ccp1 were conducted to explore the mechanism of cpd I formation. Two different mechanisms, namely the water-assisted (wet) and no-water (dry) mechanisms previously proposed, in three possible spin states (doublet, quartet, and sextet) have been investigated. The overall mechanism proceeds through two main stages; the first is the formation of hydroperoxide containing intermediate (Cpd 0) which decomposes toward ferryl-intermediate (Cpd I) in the second stage. All the geometries for the studied six reactions have been fully characterized and analyzed from a structural and energetic perspectives.

Our calculations demonstrated that the inclusion of a bridged water molecule to facilitate the reaction mechanism, did not result in significantly lowering the activation energy for the first step as suggested previously. The productive positioning of the H_2_O_2_ in RC of the dry model relative to both H52 residue and Fe(iii) centre demonstrate the unnecessarily of a water molecule to assist this process. This observation is further supported by obtaining low free energy barrier for the associated step. The dry mechanism in the doublet spin sate resulted in low energy barriers and therefore, highlight its kinetic and thermodynamic preference.

Due to the thermodynamic stability of doublet Cpd 0 intermediates (IC1 and IC2) over its RC together with high kinetic feasibility of first step of the reaction, we suggest the Cpd 0 (^2^IC1) to be the initial reacting complex for the doublet state mechanism in the direct pathway. The geometry of the calculated Cpd I agrees structurally with available crystal structures and the doublet state is the most stable. Our data also support the notion that Cpd 0 and Cpd I exist in the doublet spin state and Fe=O bond has a double-bond character at all the considered models, spin states and in presence of various dielectric media. The profound roles of the W51 and R48 distal residues in the chemical reactivity of Ccp1 have been also clarified. The geometries of the two residues are within tight H-bond distances to H_2_O_2_ and the oxo-ferly group and thus contribute to the charge neutralization developed over the transition states. Moreover, the R48 residue dictates the proper positioning of the H_2_O_2_ for the reaction to proceed.

Our investigation was further expanded to reveal the impact of the hem pocket residues in the mechanism of Cpd I formation by exploring the mechanistic, structural, and electronic behaviour of various *in silico* generated Ccp1 variants. Relative to the Gibbs energy barrier of WT Ccp1, the calculated barriers of the Ccp1 mutants are lower indicating the significance of the substituted residues in the overall mechanism. The role of these residues in obtaining a distinctive Trp π-cation radical was also explored by displaying the radical distributions for the Cpd I of the studied variants. Globally, our findings should pave the road toward further investigation of the feasibility of the proposed dry mechanism in other peroxidases due to conserved structure of the heme pocket amongst a wide range of peroxidases.

## Conflicts of interest

I declare that there is no conflict of interest.

## Abbreviations

Ccp1: Cytochrome *c* peroxidase
Cpd0: Compound 0 intermediate
Cpd1: Compound I intermediate
RC: Reactive complex
IC: Intermediate complex
TS: Transition state
PC: Product complex

## Supplementary Material

RA-012-D2RA01073A-s001
