# Supplementary material for: Mechanistic insights into the chemistry of compound I formation in heme peroxidases: quantum chemical investigations of cytochrome c peroxidase
Source: RSC Adv. 2022 May 23;12(24):15543–54. doi: 10.1039/d2ra01073a (PMC9125774; doi:10.1039/d2ra01073a)
Supplement: RA-012-D2RA01073A-s001 [file RA-012-D2RA01073A-s001.pdf]

Electronic Supplementary Material (ESI) for RSC Advances.
This journal is © The Royal Society of Chemistry 2022

Mechanistic insights into the chemistry of compound I formation in heme peroxidases: Quantum Chemical investigations of cytochrome c peroxidase

SUPPLEMENTARY INFORMATION:

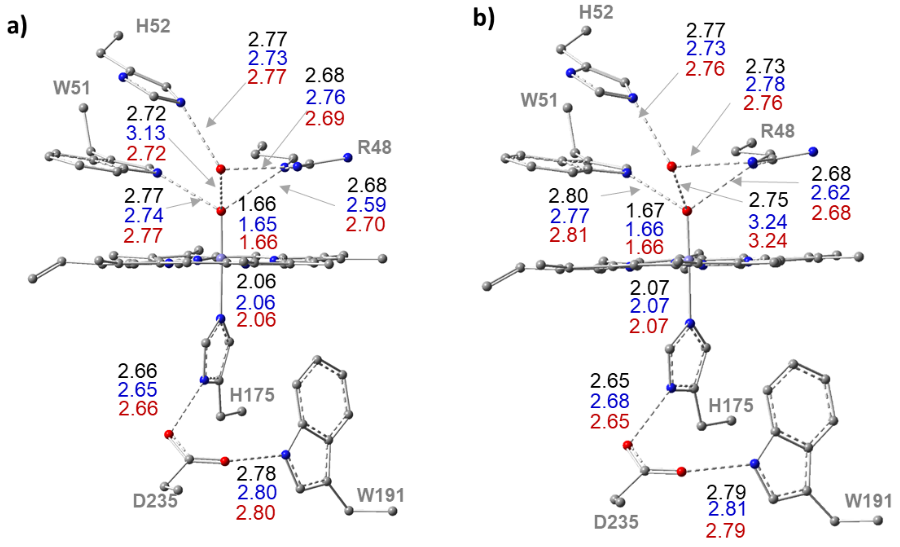

Figure S1: Cluster structures of Cpd I complexes, with selected distances between heavy atoms (Å), for three different spin states: doublet (black), quartet (blue) and sextet (red); geometries optimized with a) UB3LYP-D3BJ/6-31G(d,p) and b) UB3LYP-D3BJ/6-31+G(d,p).

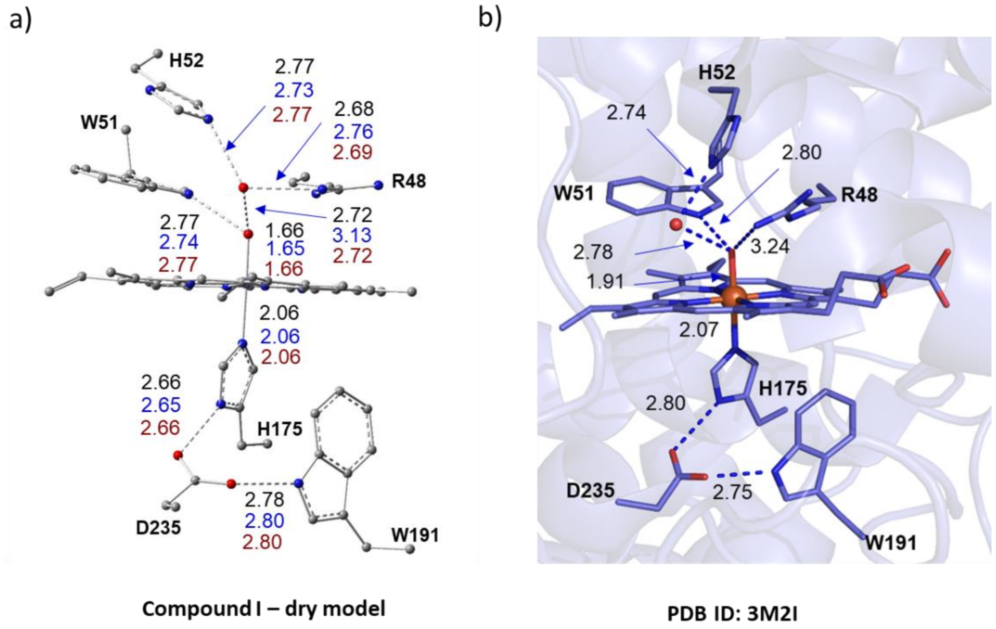

Figure S2: Cluster structures of Cpd I complexes, with selected distances between heavy atoms (Å), for a) three different spin states: doublet (black), quartet (blue) and sextet (red); b) the heme pocket of crystalized ferryl-intermediate Ccp1.

Table S1: Gibbs Free-energy (kcal/mol) for both the wet and dry mechanisms of Cpd I formation for three different spin states: Free energies are estimated with B3LYP-GD3BJ/6-311+G(2d,2p)+ΔG with ε=4.

	**Wet-mechanism**			**Dry-Mechanism**		
	**Doublet**	**Quartet**	**Sextet**	**Doublet**	**Quartet**	**Sextet**
**RC**	0.0	0.0	0.0	0.0	0.0	0.0
**TS1**	1.0	16.6	2.1	3.0	0.7	0.0
**IC1**	8.5	18.3	6.8	-5.8	0.7	1.0
**IC2**	1.4	18.6	9.0	-2.8	14.6	2.8
**TS2**	11.8	25.0	10.5	7.6	31.1	25.6
**PC**	-18.5	-17.8	-9.7	-28.4	-21.9	-14.8

Table S2: Relative-energy (kcal/mol) for both the wet and dry mechanisms of Cpd I formation for three different spin states: Free energies are estimated with UB3LYP-GD3BJ/6-311+G(2d,2p) with ε=4.

	**Wet-mechanism**			**Dry-Mechanism**		
	**Doublet**	**Quartet**	**Sextet**	**Doublet**	**Quartet**	**Sextet**
**RC**	0.0	0.0	0.0	0.0	0.0	0.0
**TS1**	1.8	19.5	4.1	3.9	1.1	1.0
**IC1**	5.4	13.4	-2.0	-1.7	1.0	0.2
**IC2**	-4.4	11.9	-2.5	-8.7	11.6	0.7
**TS2**	8.4	20.4	42.7	7.7	30.6	27.4
**PC**	-16.9	-21.5	-13.4	-26.6	-20.5	-14.6

Table S3: Relative-energy (kcal/mol) for the dry mechanisms of Cpd I formation in doublet spin states: Electronic and Free energies are estimated with BS1 (UB3LYP-GD3BJ/6-311+G(2d,2p) with ε=4) and BS2 (UB3LYP-GD3BJ/6-311+G(2df,2p).

	BS1		BS2	
	**Electronic**	**Gibbs**	**Electronic**	**Gibbs**
**RC**	0.0	0.0	0.0	0.0
**TS1**	3.9	3.0	3.9	3.0
**IC**	-1.7	-0.3	-1.7	-0.4
**IC2**	-8.7	-2.8	-8.7	-2.7
**TS2**	7.7	7.7	8.0	8.0
**PC**	-26.6	-28.4	-26.5	-28.3

Table S4: Relative Free-energy (kcal/mol) for both cpd0 and Cpd I calculated in three different spin states estimated with UB3LYP-GD3BJ/6-311+G(2d,2p) in four different dielectric constants.

	Cpd 0			Cpd I		
	Doublet	Quartet	Sextet	Doublet	Quartet	Sextet
**ε =4**	1.3	0.0	5.1	0.0	5.3	13.8
**ε =8**	2.7	0.0	7.3	0.0	9.9	14.5
**ε =16**	0.0	15.6	3.7	0.0	9.0	13.8
**ε =78.4**	0.0	16.2	3.0	0.0	2.5	13.5

Table S5: Selected distances between heavy atoms (Å) obtained for Cpd 0, for three different spin states: doublet (black), quartet (blue) and sextet (red). Geometries are optimized with UB3LYP-D3BJ/6-31G(d,p).

	Fe-O1	1O···1N_{R48}	2O···2N_{R48}	1O···N_{W51}	1O···O2	2O···N_{H52}	Fe–$N2_{H175}$	$_{H175}$N2···O_{D235}	$_{W191}$N···O_{D235}
**ε =1**	1.97	2.65	2.87	2.76	1.83	2.74	1.88	2.68	2.86
	1.97	2.67	2.88	2.77	1.76	2.76	1.89	2.67	2.86
	2.31	2.59	2.90	2.73	1.74	2.72	1.98	2.66	2.87
**ε =4**	1.93	2.72	2.87	2.77	1.48	2.78	1.91	2.62	2.85
	2.31	2.62	2.93	2.72	1.48	2.78	2.08	2.60	2.85
	2.22	2.66	2.88	2.72	1.47	2.75	2.03	2.60	2.86
**ε =8**	1.92	2.73	2.87	2.77	1.48	2.87	1.92	2.61	2.86
	1.92	2.73	2.87	2.77	1.48	2.79	1.92	2.61	2.86
	2.21	2.72	2.88	2.66	1.47	2.76	2.03	2.60	2.86
**ε =16**	1.92	2.75	2.87	2.77	1.48	2.81	1.92	2.61	2.86
	1.93	2.75	2.88	2.78	1.47	2.82	1.93	2.61	2.86
	2.21	2.67	2.88	2.72	1.47	2.76	2.04	2.60	2.85
**ε =78.4**	1.91	2.76	2.86	2.77	1.47	2.81	1.93	2.60	2.86
	1.92	2.76	2.88	2.78	1.47	2.82	1.94	2.61	2.86
	2.19	2.68	2.89	2.73	1.47	2.76	2.05	2.58	2.84

Table S6: Selected distances between heavy atoms (Å) obtained for Cpd I, for three different spin states: doublet (black), quartet (blue) and sextet (red). Geometries are optimized with UB3LYP-D3BJ/6-31G(d,p).

	Fe-O1	1O···1N$_{R48}$	2O···2N$_{R48}$	1O···N$_{W51}$	1O···O2	2O···N$_{H52}$	Fe–N2$_{H175}$	$_{H175}$N2···O$_{D235}$	$_{W191}$N···O$_{D235}$
	1.97	2.65	2.87	2.76	1.83	2.74	1.88	2.68	2.86
**ε =1**	1.97	2.67	2.88	2.77	1.76	2.76	1.89	2.67	2.86
	2.31	2.59	2.90	2.73	1.74	2.72	1.98	2.66	2.87
	1.93	2.72	2.87	2.77	1.48	2.78	1.91	2.62	2.85
**ε =4**	2.31	2.62	2.93	2.72	1.48	2.78	2.08	2.60	2.85
	2.22	2.66	2.88	2.72	1.47	2.75	2.03	2.60	2.86
	1.92	2.73	2.87	2.77	1.48	2.87	1.92	2.61	2.86
**ε =8**	1.92	2.73	2.87	2.77	1.48	2.79	1.92	2.61	2.86
	2.21	2.72	2.88	2.66	1.47	2.76	2.03	2.60	2.86
	1.92	2.75	2.87	2.77	1.48	2.81	1.92	2.61	2.86
**ε =16**	1.93	2.75	2.88	2.78	1.47	2.82	1.93	2.61	2.86
	2.21	2.67	2.88	2.72	1.47	2.76	2.04	2.60	2.85
	1.91	2.76	2.86	2.77	1.47	2.81	1.93	2.60	2.86
**ε =78.4**	1.92	2.76	2.88	2.78	1.47	2.82	1.94	2.61	2.86
	2.19	2.68	2.89	2.73	1.47	2.76	2.05	2.58	2.84

Table S7: Selected distances between heavy atoms (Å) obtained for the structures of cpd0 (IC2), for WT Ccp1 and the explored in-silico generated variants. Geometries are optimized with UB3LYP-D3BJ/6-31G(d,p).

	Fe-O1	1O···1N$_{R48}$	2O···2N$_{R48}$	1O···N$_{W51}$	1O···O2	2O···N$_{H52}$	Fe–N2$_{H175}$	$_{H175}$N2···O$_{D235}$	$_{W191}$N···O$_{D235}$
**WT**	1.99	2.76	2.81	--	1.48	2.69	1.90	2.70	2.85
**W51A**	1.97	2.74	2.85		1.48	2.55	1.91	2.72	2.86
**R48A**	1.93	--	--	2.81	1.47	2.70	2.09	2.57	2.84
**W51F/ W191F**	2.00	2.71	2.85	--	1.48	2.71	1.90	2.69	--
**W191Y**	2.01	2.76	2.82	--	1.48	2.91	1.89	2.63	2.75
**W191A**	1.97	2.74	2.85	--	1.48	2.87	1.88	2.70	--
**D235H**	1.92	2.79	2.82	--	1.48	2.84	1.95	2.82	--
**D235N**	1.95	2.76	2.84	--	1.48	3.01	1.93	2.74	--
**D235E**	1.97	2.77	2.83	--	1.48	2.81	1.92	2.62 (O1)	2.72 (O1)
**D235A**	1.90	2.81	2.85	--	1.47	2.69	1.95	--	--

Table S8: Selected distances between heavy atoms (Å) obtained for the structures of cpd0 (IC2), for WT Ccp1 and the explored variants Geometries are optimized with UB3LYP-D3BJ/6-31G(d,p).

	Fe-O1	$1O^{\cdots}1N_{R48}$	$2O^{\cdots}2N_{R48}$	$1O^{\cdots}N_{W51}$	$1O^{\cdots}O2$	$2O^{\cdots}N_{H52}$	$Fe^{\cdots}N2_{H175}$	$_{H175}N2^{\cdots}O_{D235}$	$_{W191}N^{\cdots}O_{D235}$
**WT**	1.97	2.65	2.87	2.76	1.48	2.74	1.88	2.68	2.86
**W51A**	1.95	2.69	2.88	--	1.49	2.61	1.91	2.70	2.85
**R48A**	1.90	--	--	2.76	1.48	2.65	1.93	2.62	2.83
**W51F/ W191F**	1.95	2.60	2.91	2.71	1.48	2.71	1.89	2.66	--
**W191Y**	1.97	2.65	2.90	2.76	1.48	2.76	1.88	2.61	2.74
**W191A**	1.96	2.63	2.87	2.77	1.50	2.73	1.88	2.71	--
**D235H**	1.91	2.70	2.94	2.79	1.48	2.87	1.94	2.82	--
**D235N**	1.91	2.68	2.91	2.79	1.48	2.80	1.93	2.74	--
**D235E**	1.94	2.66	2.90	2.76	1.48	2.75	1.91	2.64	--
**D235A**	1.91	2.70	2.94	2.81	1.48	2.88	1.95	--	--

TableS 9: Selected distances between heavy atoms (Å) obtained for the structures of TS2, for WT Ccp1 and the explored variants Geometries are optimized with UB3LYP-D3BJ/6-31G(d,p).

	Fe-O1	$1O^{\cdots}1N_{R48}$	$2O^{\cdots}2N_{R48}$	$1O^{\cdots}N_{W51}$	$1O^{\cdots}O2$	$2O^{\cdots}N_{H52}$	$Fe–N2_{H175}$	$_{H175}N2^{\cdots}O_{D235}$	$_{W191}N^{\cdots}O_{D235}$
**WT**	2.77	2.67	2.77	2.77	1.83	2.53	1.92	2.70	2.85
**W51A**	1.74	2.72	2.78	--	1.83	2.52	1.94	2.71	2.85
**R48A**	1.76	--	--	2.82	1.74	2.55	1.93	2.59	2.90
**W51F/ W191F**	1.74	2.62	2.79	2.59	1.84	2.59	1.92	2.68	--
**W191Y**	1.77	2.66	2.78	2.77	1.81	2.77	1.91	2.63	2.71
**W191A**	1.77	2.69	2.75	2.85	1.82	2.59	1.91	2.72	--
**D235H**	1.71	2.71	2.82	2.80	1.86	2.80	1.99	2.83	--
**D235N**	1.71	2.71	2.83	2.79	1.86	2.70	1.99	2.74	--
**D235E**	1.74	2.79	2.67	2.77	1.84	2.63	1.95	2.62	2.74(N1)
**D235A**	1.71	2.72	2.82	2.82	1.86	2.71	2.00	--	--

Table S10*:* Selected distances between heavy atoms (Å) obtained for Cpd I, for WT Ccp1 and the explored variants Geometries are optimized with UB3LYP-D3BJ/6-31G(d,p).

	Fe-O1	$1O\cdots1N_{R48}$	$2O\cdots2N_{R48}$	$1O\cdots N_{W51}$	$1O\cdots O2$	$2O\cdots N_{H52}$	$Fe{-}N2_{H175}$	${}_{H175}N2\cdots O_{D235}$	${}_{W191}N\cdots O_{D235}$
**WT**	1.66	2.68	2.68	2.77	2.72	2.77	2.06	2.66	2.78
**W51A**	1.65	2.70	2.66	--	2.74	2.76	2.09	2.67	2.85
**R48A**	1.70	--	--	2.81	2.29	2.78	1.97	2.60	2.92
**W51F/ W191F**	1.64	2.67	2.69		2.68	2.82	2.09	2.80	--
**W191Y**	1.66	2.66	2.72	2.80	2.70	2.81	2.06	2.71	2.71
**W191A**	1.67	2.71	2.66	2.79	2.72	2.74	2.06	2.80	--
**D235H**	1.67	2.74	2.70	2.66	2.73	2.75	2.05	2.86	--
**D235N**	1.67	2.74	2.70	2.66	2.73	2.75	2.05	2.77	--
**D235E**	1.67	2.66	2.71	2.72	2.70	2.82	2.03	2.65	2.59 (N1)
**D235A**	1.67	2.74	2.69	2.67	2.73	2.75	2.06	--	--

Table S11: Spin density sum Values for the major spin contaminated components of Cpd I obtained for WT Ccp1 and its studied variants calculated with UB3LYP-D3BJ/6-31G(d,p).

	**WT**	**R48A**	**W51A**	**W191Y**	**W191A**	**D235H**	**D235N**	**D235E**	**D235A**	**W51F/ W191F**
π_{FeO}	2.11	2.07	2.07	2.07	2.07	2.08	2.08	2.08	2.08	0.96
π_{His}	-0.49	0.51	-0.60	-0.66	-0.62	--	--	--	--	-0.88
π_{Trp}	-0.45	-0.21	-0.41	-0.24	--	--	-0.14	-0.55	--	--
π_{por}	**--**	-0.29	--	--	-0.36	-0.35	-0.34	-0.30	-0.38	-0.17
π_{W51}	--	0.12	--	--	-0.12	-0.65	-0.61	-0.25	-0.66	--
